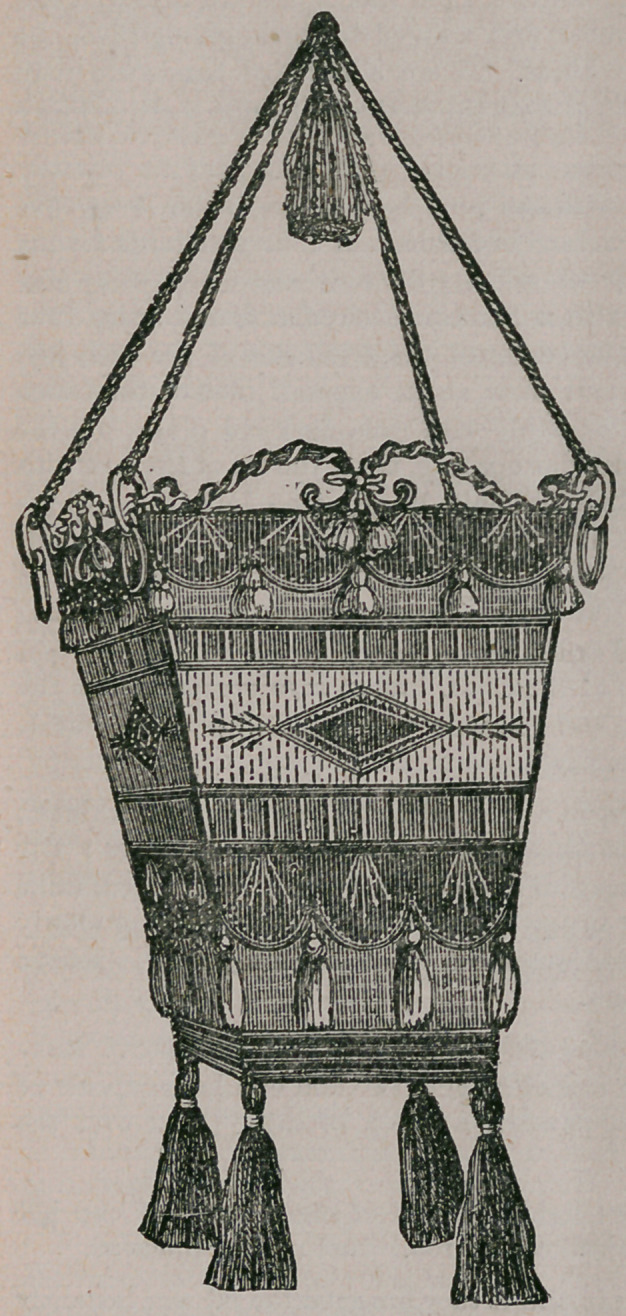# Household

**Published:** 1888-05

**Authors:** 


### HOUSEHOLD.

The above cut represents a very useful and ornamental hanging basket easily made. The basket is wicker-work, and the band at the top is of light-blne cloth four inches deep, with a scalloped piece of darker shade over it. The long stitches on the dark cloth are of the lightest shade of blue silk, with a silver threat running with it. Through the wickers run satin ribbon. Combine the two shades of blue in the tassels, with the silver wound round the tops of them. Heavy cord and tassels to hang it up by. The same design of trimming will answer for any shaped basket.

HANDSOME FOILAGE BED.—A foilage bed growing, last summer, on the grounds of Ex-Governor Weston, at Manchester, N. H., in charge of the gardener, Michael Lyon, was much admired. It was a circular bed nine feet in diameter. In the center was a Ricinus, or Castor Bean plant ten feet high, and around it were three brown Cannas, and a second circle of six green Cannas, and another circle of eight plants of Caladium esculentum. One leaf of these last named plants measured forty-one inches in length and thirty-one in breadth, the plant on which it grew standing over five feet high. One of the plants had eleven large leaves. The bed was made up preparatory to planting with mixed loam and rotted manure. —*Vick's Magazine* for April.

DWARF APPLE TREES.—A pretty thing in a garden is a nicely trained young dwarf Apple tree, or a row of them. They can be led into any desired shape, and it is lasting amusement and recreation to the amateur gardener to guide them into fanciful forms which does not debar them from giving him enjoyable fruit, always handsomer and finer than is usual on large trees. The sap has not far to travel painfully through thousands of cells and against gravity to reach the leaves from the root points, and so the leaves are completer and the fruit better fed than on the big trees.

It used to be common in the neat French gardens, and probably is yet, to see rows of dwarf Apple trees trained like low horizontal fences at the back of flower borders, separating them from the vegetable ground. In other places they would stand here and there at intervals in the borders, their shoots pinched into pyramid form or left long, but reduced in number and trained to wires, giving them the shape of letters or figures of different kinds. To an admirer of handsome fruit nothing of the kind can be more delightful than the products of these trees.—*Vick's Magazine* for April.

A Handsome Ornament.—Take a common pine box twelve inches long, five inches high, use your own ingenuity to turn or fashion out four standards for the corners about one inch high. Purchase an ordinary little circular clock—they may be found for $1, but for $2 find one with a handsome circular frame around the face—saw out a circular piece from the center of the front side of the box, just large enough to admit the clock face and show about one-half inch of the frame around the face ; then cover the box smoothly with rich, dark red plush, put the clock in the box with the face in the circular opening, cover the lid of the box with the plush and fasten on. Have ready some molding about an inch wide, which may be procured at a trifling cost from any picture frame dealer ; bronze the molding carefully with any good bronzing powder, then glue the molding firmly around the edges of the box and bronze the standards. Now, to beautify the clock, find some plaster of Paris statuette, eight or ten inches long, four or fiveinches wide, and not too high to look well on top of the clock, bronze it to compare with the molding, and place on top of the box, and you have a handsome parlor ornament. —*Decorator and Furnisher.*

Vegetable Soup.—Put into a saucepan a piece of butter the size of a walnut ; when it is very hot put in three onions sliced and a half-dozen celery leaves ; stir until they redden, then add a half-teacupful of flour and when this is red, (take great care that it does not burn), pour in one pint of boiling water, stirring slowly all the while, then add one quart of cold water and simmer for an hour. Season with salt and pepper and serve, very hot.

Bird's Nest Pudding.—Pare four good-sized sour spples, stew until soft. Make a batter of one cup of milk, butter the size of an egg, two and one half cupfuls of flour, two heaping teaspoonfuls baking powder, a pinch of salt. Pour over the stewed apples and bake in a hot oven.

*Sauce for the Above.*—One egg beaten light, one cup of sugar, one-half cup hot water, one sliced lemon, one tablespoonful cornstarch. Boil until it thickens.

To test nutmegs prick them with a pin and if they are good the oil will instantly spread around the puncture.

Hams wrapped in thick brown paper and packed in a barrel of wood ashes in the cellar, will keep all summer.

All fish skin should be washed thoroughly, cut in small bits and put in a box or paper bag, to use in settling coffee.

## Figures and Tables

**Figure f1:**